# Pyruvate Administration Restores Impaired Nociception by Enhancing Neurite Outgrowth in Streptozotocin-Induced Diabetic Mice

**DOI:** 10.3390/ijms26104666

**Published:** 2025-05-13

**Authors:** Hideji Yako, Mari Suzuki, Shizuka Takaku, Naoko Niimi, Ayako Kato, Koichi Kato, Junji Yamauchi, Kazunori Sango

**Affiliations:** 1 Diabetic Neuropathy Project, Tokyo Metropolitan Institute of Medical Science, Tokyo 156-8506, Japantakaku-sz@igakuken.or.jp (S.T.); niimi-nk@igakuken.or.jp (N.N.); yamauchi@toyaku.ac.jp (J.Y.); 2 Laboratory of Molecular Neuroscience and Neurology, Tokyo University of Pharmacy and Life Sciences, Tokyo 192-0982, Japan; 3 Laboratory of Medicine, School of Pharmacy, Aichi Gakuin University, Nagoya 470-0195, Japan; k-ayako@dpc.agu.ac.jp (A.K.); kkato@dpc.agu.ac.jp (K.K.); 4 Laboratory of Molecular Pharmacology, National Research Institute for Child Health and Development, Tokyo 157-8535, Japan

**Keywords:** pyruvate, diabetic peripheral neuropathy, dorsal root ganglion neurons, nociception

## Abstract

Diabetic peripheral neuropathy (DPN) is a chronic complication of diabetes mellitus for which effective treatments remain undeveloped. Metabolic changes and inflammation are proposed as primary mechanisms underlying DPN pathogenesis. Our previous studies demonstrate that exogenous pyruvate plays a crucial role in maintaining glycolysis-tricarboxylic acid cycle flux under high-glucose conditions and also exhibits anti-inflammatory properties. To evaluate its therapeutic potential, we assessed whether pyruvate administration could restore DPN in vivo and in vitro. We assessed casual blood glucose levels, body weight, motor and sensory nerve conduction velocities, mechanical sensitivity, and intraepidermal nerve fiber density in streptozotocin-induced diabetic C57/BL/6J mice that received drinking water with or without sodium pyruvate (10 mg/mL) from 2 to 13 weeks after diabetes induction. In addition, we evaluated neurite length in ND7/23 cells, a dorsal root ganglion neuron cell line, under high-glucose conditions. Pyruvate administration in diabetic mice alleviated mechanical sensitivity deficits and improved intraepidermal nerve fiber density. Additionally, neurite length in ND7/23 cells was inhibited under high-glucose conditions but was fully restored by supplementation with high concentrations (10 mM) of pyruvate. These findings suggest that exogenous pyruvate may be a promising therapeutic candidate for DPN.

## 1. Introduction

Diabetic peripheral neuropathy (DPN), along with retinopathy and nephropathy, is one of the chronic complications of diabetes mellitus and affects approximately 36% of patients with type 2 diabetes in Japan [1]. The pathogenesis of DPN is attributed to diabetes-induced metabolic changes in dorsal root ganglion (DRG) neurons, Schwann cells, and vascular endothelial cells in the peripheral nervous system. These changes lead to the formation of advanced glycated endproducts, oxidative stress, endoplasmic reticulum stress, inflammation, and mitochondrial damage [2,3,4,5]. These lesions ultimately result in symmetric, length-dependent distal axonal degeneration and sensory impairment. Despite extensive research, no effective therapies targeting the underlying mechanisms of DPN have been developed to date. 

Omics studies on DPN have demonstrated that dysmetabolism and inflammation in DRGs and sciatic nerves contribute significantly to the development and progression of DPN [6,7,8]. Exogenous pyruvate, known for its regulatory effects on glucose metabolism [9,10] and anti-inflammatory properties [11], has been identified as a potential therapeutic candidate for DPN. However, serum pyruvate levels under diabetic conditions remain inconsistent across studies. While some findings indicate elevated serum pyruvate in diabetes [12,13,14], others report reduced serum pyruvate levels in both streptozotocin (STZ)-induced diabetic rats [15,16,17,18] and patients with type 2 diabetes [19], particularly among those with diabetic complications [19]. 

Our previous studies revealed that exogenous pyruvate starvation led to cultured Schwann and neuronal cell death under high-glucose conditions [9]. These cells experienced necrosis-like death caused by ATP depletion [10], highlighting the critical role of exogenous pyruvate in maintaining the flux in glycolysis, glycolytic collateral pathways, tricarboxylic acid (TCA) cycle, and ATP production. However, the potential of pyruvate administration to prevent or restore DPN in diabetic mice remains unclear. Pyruvate administration to diabetic animals has been shown to restore hyperglycemia [20], diabetic retinopathy [21,22], and diabetic nephropathy [20,23]. Therefore, we assessed the efficacy of exogenous pyruvate in preventing and restoring DPN using diabetic models in vivo and in vitro.

## 2. Results

### 2.1. Pyruvate Administration Ameliorated Impaired Nociception in STZ-Induced Diabetic Mice

**Figure 1:**
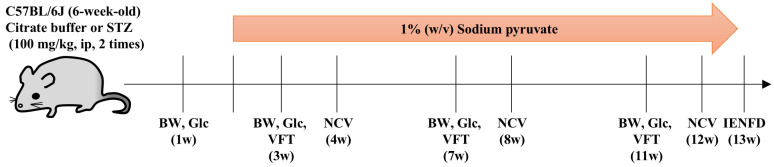
Schematic representation of the animal study. Six-week-old mice were injected with streptozotocin (STZ) or citrate buffer. Following injection, the mice were provided ad libitum access to drinking water, in the presence or absence of sodium pyruvate, from 2 to 13 weeks post-injection. The experiments were conducted as shown in this figure. BW: body weight, Glc: casual blood glucose, VFT: von Frey test, NCV: motor and sensory nerve conduction velocity, IENFD: intraepidermal nerve fiber density.

This study examined the therapeutic efficacy of pyruvate administration on DPN using STZ-induced diabetic mice, as pyruvate administration has been shown to restore blood glucose levels in db/db mice, a model of type II diabetes [20]. The mice were divided into four groups: control mice receiving water (CW, *n* = 8), control mice receiving a pyruvate solution (CP, *n* = 7), STZ mice receiving water (SW, *n* = 8), and STZ mice receiving a pyruvate solution (SP, *n* = 8) (Figure 1). 

**Figure 2:**
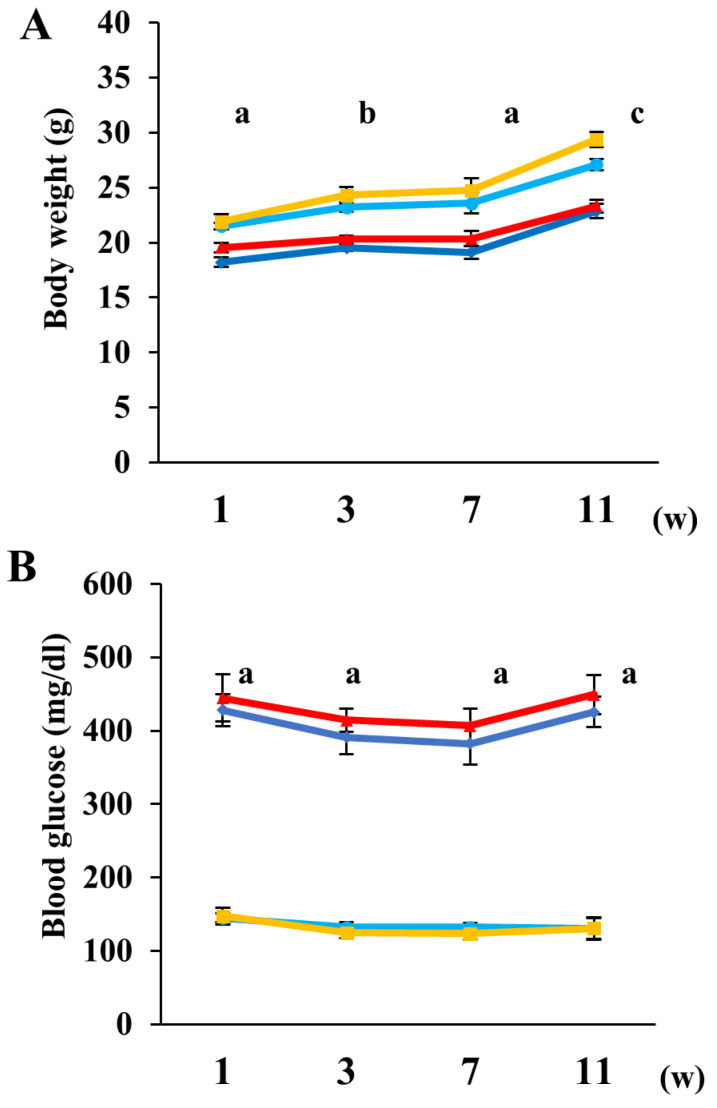
Pyruvate administration did not affect body weight or blood glucose levels in both control and STZ-injected mice. Body weight (**A**) and casual blood glucose levels (**B**) were measured at 1, 3, 7, and 11 weeks after STZ injection. Data are presented for buffer-injected mice drinking water (CW, light blue, *n* = 8) or pyruvate (CP, yellow, *n* = 7) and for STZ-injected mice drinking water (SW, blue, *n* = 8) or pyruvate (SP, red, *n* = 8). Values represent the mean ± SEM. Statistical analysis was performed using one-way analysis of variance (ANOVA), followed by post hoc comparisons with the Tukey HSD test. (**A**) a: *p* < 0.01 CW vs. SW and CP vs. SW, *p* < 0.05 CW vs. SP, and CP vs. SP, b: *p* < 0.01 CW vs. SW, CW vs. SP, CP vs. SW and CP vs. SP; and, c: *p* < 0.01 CW vs. SW, CP vs. SW and CP vs. SP, *p* < 0.05 CW vs. SP. (**B**) a: *p* < 0.01 CW vs. SW, CW vs. SP, CP vs. SW and CP vs. SP.

At all observed time points, the SW and SP groups exhibited significantly lower body weights and higher blood glucose levels compared to the CW and CP groups. In contrast, no significant differences in body weight or blood glucose levels were observed between the CW and CP groups, nor between the SW and SP groups (Figure 2). These results indicate that pyruvate supplementation did not alter body weight and blood glucose levels in either control or diabetic mice. 

**Figure 3:**
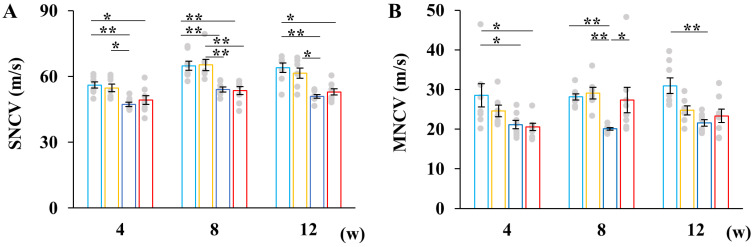
Pyruvate administration to STZ-induced diabetic mice did not restore sensory and motor nerve conduction velocities. Sensory nerve conduction velocity (SNCV; (**A**) and motor nerve conduction velocity (MNCV; (**B**) were measured at 4, 8, and 12 weeks after STZ injection. Data are presented for buffer-injected mice drinking water (CW, light blue, *n* = 8) or pyruvate (CP, yellow, *n* = 7) and for STZ-injected mice drinking water (SW, blue, *n* = 8) or pyruvate (SP, red, *n* = 8). Values represent the mean ± SEM with individual values depicted as circles. Statistical analysis was performed using the *Kruskal-Willis* test, followed by post hoc comparisons with the Steel-Dwass test. * *p* < 0.05, ** *p* < 0.01.

Sensory and motor nerve conduction velocities (SNCV and MNCV, respectively) and the von Frey test were used to assess DPN symptoms. SNCV in STZ-induced diabetic mice was significantly reduced at all time points compared to buffer-injected mice, with no significant differences observed between the SW and SP groups (Figure 3A). 

Although MNCV in the SW and SP groups was reduced at 4 and 12 weeks after STZ injection compared to the CW and CP groups, MNCV in the SP group was transiently improved 8 weeks after the injection. However, 12 weeks after injection, the significance was not observed. Pyruvate administration to control animals reduced MNCV, compared with CW group (Figure 3B).

**Figure 4:**
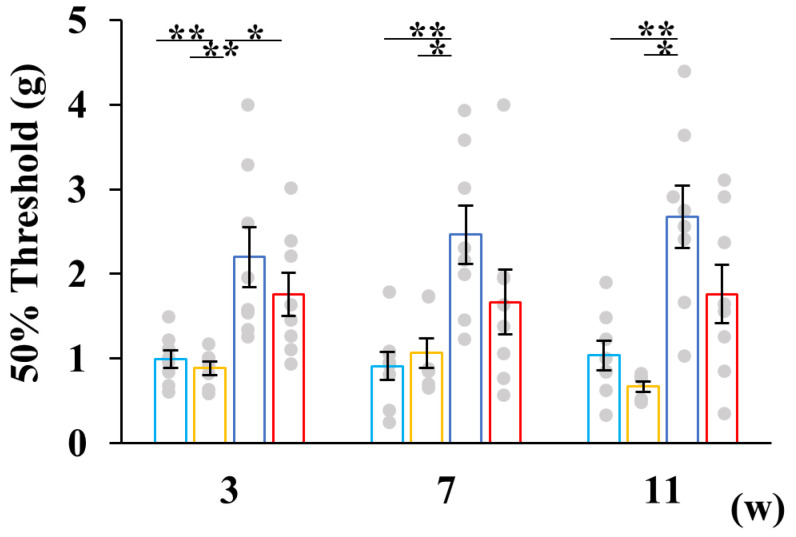
Pyruvate administration tended to improve impaired nociception in STZ-injected mice. The von Frey test was conducted at 4, 8, and 12 weeks after STZ injection. Data are presented for buffer-injected mice drinking water (CW, light blue, *n* = 8) or pyruvate (CP, yellow, *n* = 7) and for STZ-injected mice drinking water (SW, blue, *n* = 8) or pyruvate (SP, red, *n* = 8). Values represent the mean ± SEM, with individual values depicted as circles. Statistical analysis was performed using the *Kruskal-Willis* test, followed by post hoc comparisons with the Steel-Dwass test. * *p* < 0.05, ** *p* < 0.01.

Moreover, hypesthesia was observed in the SW groups, while pyruvate administration tended to restore impaired nociception (Figure 4).

**Figure 5:**
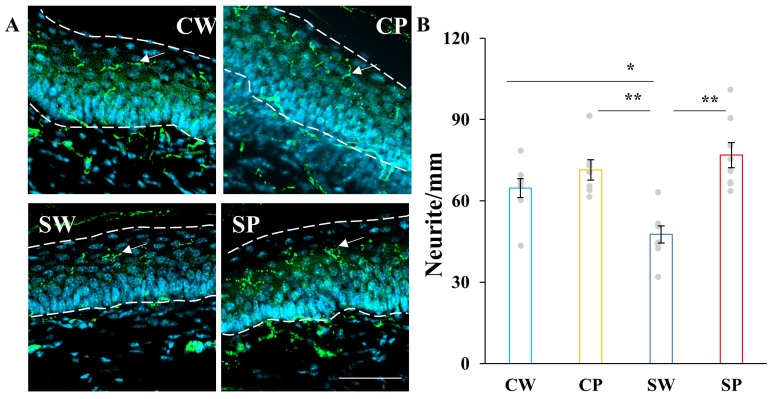
Pyruvate administration restored intraepidermal nerve fiber density (IENFD) in STZ-induced diabetic mice. IENFD (**A**,**B**) was estimated at 13 weeks after STZ injection. (**A**) Intraepidermal nerve fibers indicated by white arrows were detected by immunohistochemistry using an anti-PGP 9.5 antibody. The dot lines show epidermis. (**B**) IENFDs were quantified from the images shown (**A**). Data are presented for buffer-injected mice drinking water (CW, light blue, *n* = 8) or pyruvate (CP, yellow, *n* = 8) and for STZ-injected mice drinking water (SW, blue, *n* = 8) or pyruvate (SP, red, *n* = 8). Scale bar: 50 μm. Values represent the mean ± SEM, with individual values depicted as circles. Statistical analysis was performed using one-way ANOVA, followed by post hoc comparisons with the Tukey HSD test. * *p* < 0.05, ** *p* < 0.01.

Measurement of intraepidermal nerve fiber density (IENFD) revealed a reduction in nerve fibers in the hind paw of the SW group compared to the CW and CP groups, while pyruvate administration restored IENFD in STZ-induced diabetic mice (SP) (Figure 5). These results demonstrate that pyruvate has beneficial effects on DPN, restoring nociceptive function and improving nerve lesions.

### 2.2. Exogenous Pyruvate Enhanced the Neurite Outgrowth in Cultured DRG Neurons Under Hyperglycemia

**Figure 6:**
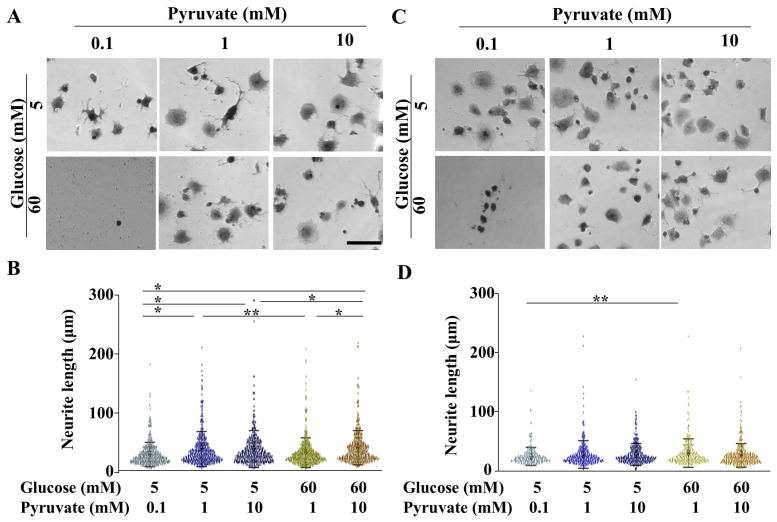
High conditions of pyruvate prevented ND7/23 cells from hyperglycemia-induced inhibition of neurite length. Differentiated (**A**) and undifferentiated (**C**) ND7/23 cells were exposed to either 5 or 60 mM glucose with 0.1, 1, or 10 mM pyruvate. Cells exposed to 60 mM glucose in the presence of 0.1 mM pyruvate experienced cell death. Neurite length was visualized using a βIII tubulin antibody. Neurite length measurements of differentiated (**B**) and undifferentiated (**D**) ND7/23 cells were taken under normal glucose conditions (5 mM, blue colors) and high glucose conditions (60 mM, yellow colors), with 0.1, 1, or 10 mM pyruvate. Scale bar: 100 μm. Box and whisker plots represent data from 199 to 366 cells across three independent experiments. Statistical analysis was performed using one-way ANOVA, followed by post hoc comparisons with the Tukey HSD test. * *p* < 0.05, ** *p* < 0.01.

ND7/23 cells, a hybrid cell line derived from mouse neuroblastoma and neonatal rat DRG neuron hybrid cells, express several DRG neuron markers [24]. Sodium pyruvate is present at concentrations of 1.1 mM in cell culture media and 0.1 mM in mouse serum. ND7/23 cells exhibited hyperglycemia-induced damage under 60 mM glucose conditions [25]. We previously reported that pyruvate starvation induces neuronal cell death under hyperglycemic conditions. Furthermore, IMS32 Schwann cells exposed to 15 mM glucose underwent complete and partial cell death at pyruvate concentrations of 0.01 mM and 0.1 mM, respectively. This pyruvate starvation-induced cell death occurred in a glucose concentration-dependent manner [9]. Based on these findings, we assumed that 0.1 mM pyruvate–equivalent to the serum level in mice–would lead to cell death under 60 mM glucose conditions; therefore, we did not perform experiments in the absence of pyruvate. Instead, we investigated the effect of pyruvate on neurite outgrowth in ND7/23 cells cultured under 5 mM or 60 mM glucose in the presence of 0.1, 1, and 10 mM pyruvate. ND7/23 cell death was observed under high-glucose (60 mM) and low-pyruvate (0.1 mM) conditions (Figure 6A,C), as well as in primary-cultured DRG neurons under high-glucose pyruvate-starved conditions [9]. Under differentiated conditions, the neurite length of ND7/23 cells exposed to normal glucose (5 mM) conditions in the presence of 1 mM exogenous pyruvate was greater than that in the presence of 0.1 mM pyruvate, and it was comparable to the neurite length in the presence of 10 mM pyruvate (Figure 6B). Exposure to high-glucose conditions with 1 mM pyruvate inhibited neurite length compared to normal glucose conditions with 1 mM pyruvate. However, this inhibition was prevented by supplementation with 10 mM pyruvate. 

Under undifferentiated conditions, exogenous pyruvate levels did not markedly affect neurite length under either normal- or high-glucose conditions (Figure 6D). These data indicate that exogenous pyruvate enhances neurite outgrowth in ND7/23 cells under both normal- and high-glucose conditions.

**Figure 7:**
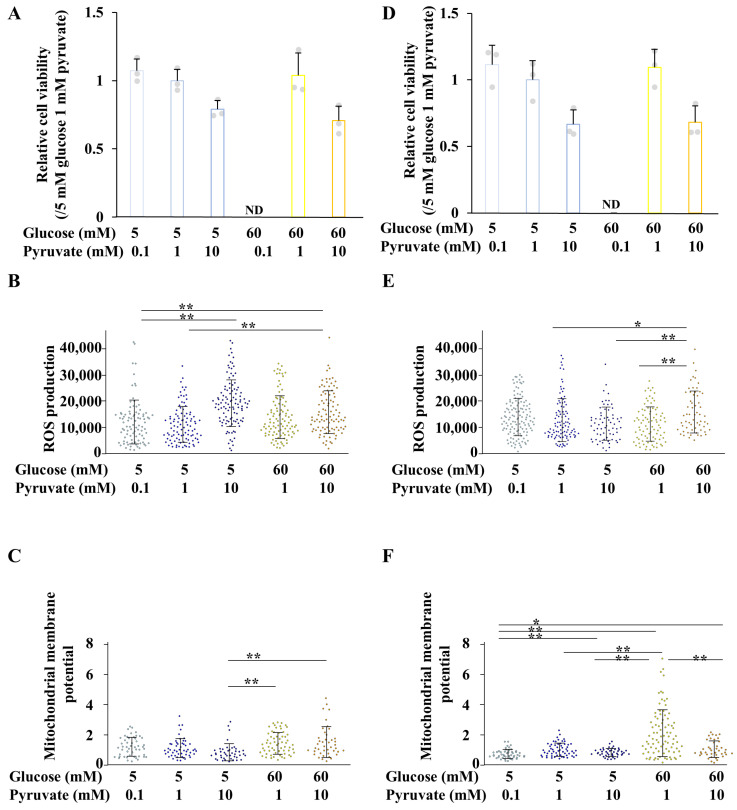
Measurement of cell viability, ROS production, and mitochondrial membrane potential of ND7/23 cells under differentiated and undifferentiated conditions. Differentiated (**A**–**C**) and undifferentiated (**D**–**F**) ND7/23 cells were exposed to either 5 (blue bars) or 60 mM (yellow bars) glucose with 0.1, 1, or 10 mM pyruvate. Cell viability (**A**,**D**), ROS production (**B**,**E**), and mitochondrial membrane potential (**C**,**F**) under each condition were assessed. Values represent the mean + SD, with individual values depicted as circles (**A**,**D**). Bar graph and box and whisker plots represent data from 3 wells (**A**,**D**), 61 to 125 cells (**B**,**E**) and 40 to 66 cells (**C**,**F**) across three independent experiments. Statistical analysis was performed using one-way ANOVA, followed by post hoc comparisons with the Tukey HSD test (**C**,**E**), or Kruskal-Wilis test, followed by post hoc comparisons with the Steel Dwass test (**A**,**B**,**D**,**F**). * *p* < 0.05, ** *p* < 0.01.

Cell viability of ND7/23 cells, under both differentiated and undifferentiated conditions, was reduced in high-glucose, low-pyruvate environments, but remained unchanged under other conditions (Figure 7A,D). Under differentiated conditions, ROS production was significantly increased under normal- and high- glucose conditions in the presence of 10 mM pyruvate. In contrast, mitochondrial membrane potential was reduced under normal-glucose, high-pyruvate conditions compared to high-glucose conditions (Figure 7B,C). These findings suggest that ROS production is enhanced in association with mitochondrial activation under high-glucose conditions, whereas under normal-glucose conditions, ROS production increases despite mitochondrial inhibition. Under undifferentiated conditions, ROS production tended to decrease in a pyruvate concentration-dependent manner under normal-glucose conditions, but was significantly elevated under high-glucose, high-pyruvate conditions (Figure 7E,F). Collectively, these results indicate that neurite outgrowth in ND7/23 cells at high pyruvate levels is accompanied by increased ROS production, and is not correlated with mitochondrial membrane potential.

**Figure 8:**
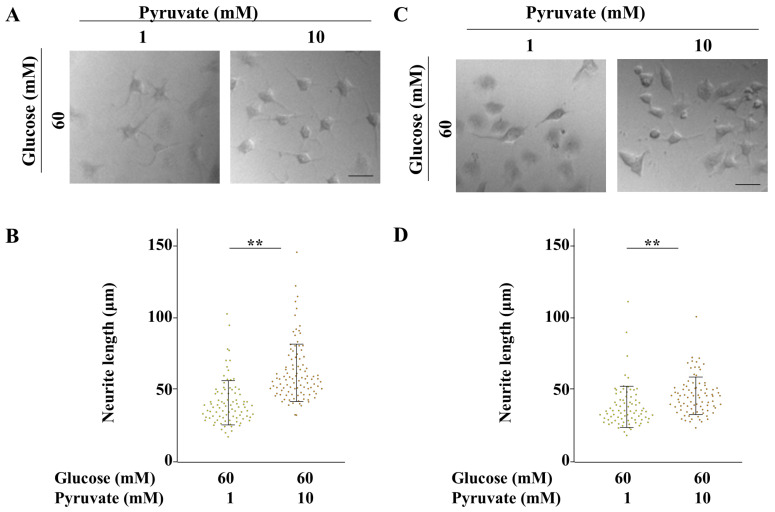
High levels of pyruvate prevented ND7/23 cells from hyperglycemia-induced neurite degeneration. Neurite outgrowth of ND7/23 cells was enhanced under differentiated conditions followed by maintenance under 60 mM glucose in the presence of 1 or 10 mM pyruvate under differentiated (**A**,**B**) and undifferentiated (**C**,**D**) conditions. Phase contrast images of ND7/23 cells under differentiated (**A**) and undifferentiated (**C**) conditions are shown. Neurite length measurements of differentiated (**B**) and undifferentiated (**D**) ND7/23 cells were taken under 60 mM glucose conditions with 1 mM (yellow dots), or 10 mM (orange dots) pyruvate. Scale bar: 100 μm. A combination of dot plots and bars of ±SD represent data from 79 to 103 cells across three independent experiments. Statistical analysis was performed using Student’s *t* test. ** *p* < 0.01.

**Figure 9:**
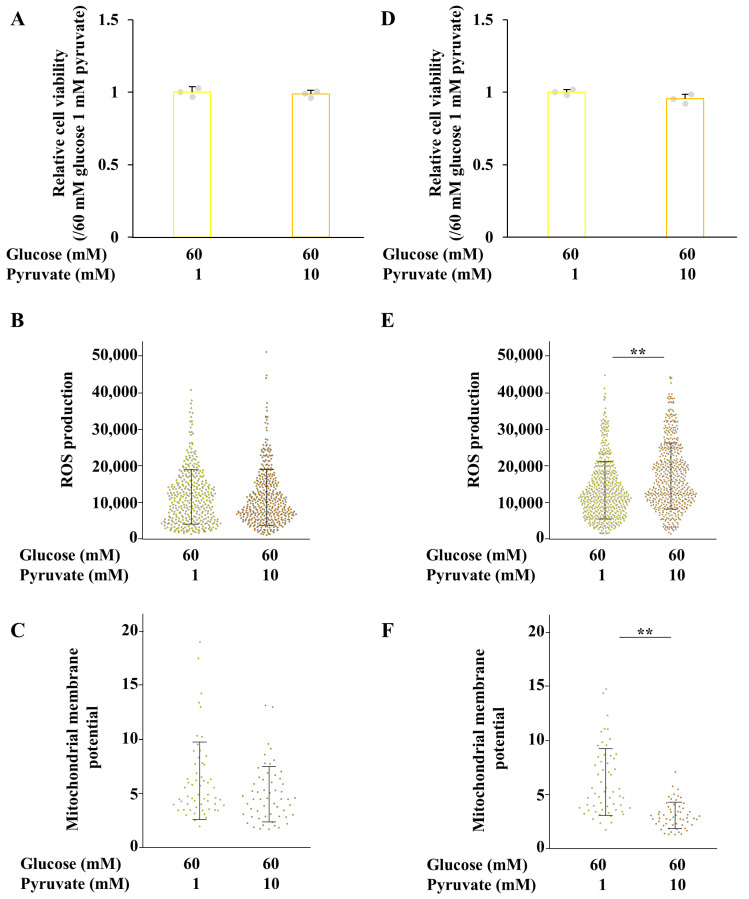
High levels of pyruvate prevented ND7/23 cells from hyperglycemia-induced neurite degeneration. Neurite outgrowth of ND7/23 cells were enhanced under differentiated conditions and then exposed to 60 mM glucose in the presence of 1 or 10 mM pyruvate under differentiated (**A**–**C**) and undifferentiated (**D**,**E**) conditions. Bar graph and box and whisker plots exhibit the data of cell viability (**A**,**D**), ROS production (**B**,**E**), and mitochondrial membrane potential (**C**,**F**) under 60 mM glucose conditions with 1 mM (yellow bars), or 10 mM (orange bras) pyruvate. Values represent the mean + SD, with individual values depicted as circles (**A**,**D**). Bar graph and box and whisker plots represent data from 3 wells (**A**,**D**), 342 to 418 cells (**B**,**E**) and 57 cells (**C**,**F**) across three independent experiments. Statistical analysis was performed using student’s *t* test. ** *p* < 0.01.

To further investigate the effect of pyruvate on neurite degeneration, ND7/23 cells were maintained under differentiated conditions and subsequently exposed to high-glucose conditions in the presence of 1 or 10 mM pyruvate, under differentiated and undifferentiated states (Figure 8 and Figure 9). In both conditions, high levels of pyruvate promoted neurite outgrowth without affecting cell viability (Figure 9A,D). Although high-pyruvate concentrations increased ROS production and impaired mitochondrial membrane potential under undifferentiated conditions, these parameters remained unchanged under differentiated conditions (Figure 9B,C,E,F). These findings demonstrate that exogenous pyruvate prevents neurite degeneration under hyperglycemic conditions.

## 3. Discussion

We previously reported that exogenous pyruvate has an essential role in maintaining glycolysis, glycolytic collateral pathways, and TCA cycle flux under high-glucose conditions in vitro [9,10]. However, the therapeutic efficacy of pyruvate on DPN in vivo remains uncertain. Therefore, this study examined the therapeutic efficacy of exogenous pyruvate on DPN using STZ-induced diabetic mice, demonstrating that pyruvate administration restored impaired nociception by enhancing neurite outgrowth in DRG neurons. Therefore, pyruvate administration represents a potential candidate for DPN therapy.

Pyruvate is converted to acetyl-CoA by pyruvate dehydrogenase (PDH), which is regulated by PDH kinase (PDK). Pdk2 and Pdk4 double knockout mice with STZ-induced diabetes reveal the attenuated DPN pathogenesis via suppressing lactate-induced inflammation [26]. Lactate-LCN2-PDK2 axis in satellite glial cells contributes to the progression of DPN [27]. These results indicate that pyruvate dysmetabolism leads to the development and progression of DPN. In the sciatic nerve of db/db mice, exogenous pyruvate metabolism was enhanced in the TCA cycle compared to db/+ mice, while glucose utilization was suppressed in glycolysis [28]. Moreover, exogenous pyruvate maintains TCA cycle flux via PDH activity in a PDK-independent pathway in IMS32 Schwann cells under high-glucose conditions [9,10]. These findings indicate that exogenous pyruvate accelerated TCA cycle flux and mitochondrial ATP production in the diabetic sciatic nerve. 

Monocarboxylate transporters (MCTs) are primarily recognized as lactate transporters [29], but MCTs also serve as pyruvate transporters [30]. Heterozygous MCT1 null mice with STZ-induced diabetes led to severe DPN, compared with those of wild-type mice [31]. Differential conditional knockout mice of Schwann cell–specific Mct1 revealed the role of MCT1 in maintaining sensory nerve myelination [32] and motor end-plate innervation [33] through lipid metabolism. Moreover, MCT1 is important for the regeneration of sensory and motor axons following sciatic nerve crush [34] and survival axons following sciatic nerve injury [35]. These findings demonstrate that MCT1-mediated transport into DRG neurons and Schwann cells plays a crucial role in maintaining and recovering the PNS. Although lactate metabolism between neurons and glia remains crucial, we believe that pyruvate metabolism as well as lactate metabolism essentially contribute to PNS homeostasis and DPN pathogenesis. 

Decreases in NCV and IENFD are major symptoms in DPN; however, pyruvate treatment restored IENFD and transiently rescued MNCV. Omics studies in pioglitazone-treated db/db mice demonstrated that metabolic alteration and inflammation induced by the diabetic condition led to lesions in small and large fibers, respectively [7,8]. Exogenous pyruvate acts as both an antioxidant and anti-inflammatory molecule, protecting against neuronal cell death induced by hyperglycemia [9] and H_2_O_2_ [36]. These findings suggest that pyruvate treatment for DPN may be sufficient to maintain glucose metabolism and insufficient to provide anti-inflammatory activity. However, inflammatory cytokines such as TNF-α and IL-1β, as well as M1 macrophage infiltration, are elevated in the sciatic nerves of 9-week-old db/db mice compared to those of 13-week-old db/db mice [37]. These findings suggest that inflammation may be transiently induced during the early stages of DPN. Based on these observations and our own data, pyruvate administration may alleviate inflammation and restore MNCV at 8 weeks after STZ injection. After 8 weeks, however, other factors—such as altered lipid metabolism and extracellular matrix homeostasis—may contribute to the progression of DPN [38]. The effects of exogenous pyruvate on ROS production and mitochondrial membrane potential in ND7/23 cells vary during processes of differentiation and degeneration. During differentiation, high levels of pyruvate increased ROS production without affecting mitochondrial membrane potential under both normal- and high-glucose conditions. In contrast, during neurite degeneration, high levels of pyruvate did not affect ROS production or mitochondrial membrane potential under high-glucose conditions. Under nondifferentiated conditions, exogenous pyruvate prevented abnormal mitochondrial membrane potential in ND7/23 cells. Taken together with results from both animal models and cultured cells, these findings suggest that exogenous pyruvate may help prevent axonal degeneration by supporting glucose metabolism without enhancing ROS production in DPN.

MNCVs were significantly reduced in control mice 12 weeks after pyruvate administration. Dimethylglyoxal, a byproduct of lactate-derived pyruvate that induces oxidative stress, is known to be elevated in diabetes [39]. Therefore, it is possible that pyruvate administration leads to the production of dimethylglyoxal, which may contribute to reduced MNCV.

This study found that exogenous pyruvate accelerated the neurite outgrowth and protected against neurite degeneration in ND7/23 cells under both normal and high-glucose conditions. Moreover, ethyl pyruvate treatment recovers the PI3K-Akt pathway, which is critical for neurite outgrowth [40], in animals with pulmonary arterial hypertension and in pulmonary artery endothelial cells under low oxygen conditions [41]. The lactate-MCT2-Akt-GSK3β pathway has also been shown to mediate neurite outgrowth in rat cortical neurons [42]. These findings indicate that pyruvate can be incorporated into neurons via MCT2, leading to activation of the Akt pathway and promotion of neurite outgrowth.

Our study demonstrates that pyruvate administration ameliorated impaired nociception and nerve fiber density in the hind paw in STZ-induced diabetic mice and enhanced neurite outgrowth of differentiated ND7/23 cells. However, two limitations in this study should be noted: (1) serum pyruvate levels in STZ-induced diabetic mice were not measured, and (2) the mechanism by which pyruvate improves nociception and neurite outgrowth, but not NCVs, remains unclear. Future studies will address these issues and explore the therapeutic efficacy of pyruvate on DPN pathogenesis in vivo and in vitro.

## 4. Materials and Methods

### 4.1. Animals

STZ-induced diabetic and citrate buffer–injected mice were obtained from Japan SLC, Inc. (Shizuoka, Japan). Diabetes was induced by a single intraperitoneal (i.p.) injection of STZ in citrate buffer at a dose of 100 mg/kg for 2 consecutive days in 6-week-old male C57BL/6J mice. Diabetes was considered present when blood glucose levels exceeded 300 mg/dL. Two weeks after STZ or citrate buffer injection, the mice received ad libitum access to drinking water, with or without sodium pyruvate (10 mg/mL) [20,21] (Sigma–Aldrich Co. LCC, St. Louis, MO, USA) until 13 weeks post-injection. These mice were categorized as control mice without (CW, *n* = 8) and with a pyruvate solution (CP, *n* = 7), and STZ mice without (SW, *n* = 8), and with a pyruvate solution (SP, *n* = 8). Body weight and casual blood glucose levels were measured at 1, 3, 7, and 11 weeks after the injection. The von Frey test and nerve conduction velocity measurements were conducted at 1, 3, 7, and 11 weeks and 4, 8, and 12 weeks, respectively (Figure 1). Mice were fed standard chow ad libitum and housed in a temperature- and humidity-controlled room with a 12:12 h light-dark cycle, in cages measuring 22 cm × 22 cm × 13.8 cm. All mice received humane care and handling in accordance with the ARRIVE guidelines. All experiments were approved by the Institutional Review Board of the Tokyo Metropolitan Institute of Medical Science (institutional approval number 20–016, 2020) and were performed in accordance with the Guidelines for the Care and Use of Animals of the Tokyo Metropolitan Institute of Medical Science (2011), 1 April 2020.

### 4.2. Nerve Conduction Velocity

Motor and sensory nerve conduction velocity (MNCV and SNCV, respectively) in the sciatic-tibial nerves were evaluated under isoflurane anesthesia, with measurements conducted on a heating pad. Recording and distal stimulating electrodes were inserted into the interosseous muscles between the second, third, and fourth toes, and near the ankle, close to the Achilles tendon, respectively. Motor nerve action potential was measured using the PowerLab system (ADInstruments Japan Inc., Nagoya, Japan). Proximal stimulating electrodes were inserted into the sciatic notch, and action potentials were recorded. For sensory nerve action potential, recording and proximal stimulating electrodes were replaced, and the distal electrodes were stimulated. MNCV was calculated based on the differences in latency between proximal and distal action potentials.

### 4.3. von Frey Test

Mice were placed on a mesh stand and allowed to acclimate for 15 to 30 min. A range of von Frey filaments (North Coast Medical, Inc., Morgan Hill, CA, USA) were used: 0.04, 0.07, 0.16, 0.4, 0.6, 1, 1.4, 2, and 4 g. Starting with the 0.6 g filament, pressure was applied to the plantar muscle of the hind paw, and the 50% threshold was estimated as the average of two trials performed with the up-down testing method and analyzed using the Up-Down Reader (UDReader, v2.0) [43]. 

### 4.4. Intraepidermal Nerve Fiber Density 

Thirteen weeks after diabetes induction, the mice were anesthetized with isoflurane, and their hind paws were dissected. The hind paws were fixed in 4% paraformaldehyde (Nacalai Tesque Inc., Kyoto, Japan) containing 50 mM phosphate buffer overnight at 4 °C, then immersed in 30% sucrose in 50 mM phosphate buffer at 4 °C for cryoprotection. The tissues were embedded in O.C.T compound (Sakura Finetek Japan Co., Ltd., Tokyo, Japan) and cryosectioned to a thickness of 50 μm. After washing with phosphate buffer saline (PBS), the cryosections were immersed in 0.4% Block Ace (DS Pharma Biomedical, Osaka, Japan) containing 0.1% TritonX 100 (blocking buffer) for permeabilization. The sections were incubated overnight at 4 °C with rabbit anti-PGP9.5 antibody (1:1000, Proteintech Group, Rosemont, IL, USA) diluted in blocking buffer. After washing with PBS, the sections were incubated for 1 h at 37 °C with chicken Alexa 488 conjugated-anti-rabbit IgG antibody (1:200; Thermo Fisher Scientific Inc., Waltham, MA, USA) diluted in PBS. The sections were mounted with DAPI Fluoromount-G (Southern Biotech, Birmingham, AL, USA). Fluorescent images were captured using a confocal laser scan microscope (LSM780, Carl Zeiss, Jena, Germany).

### 4.5. Cell Culture and Differentiation

ND7/23 cells were generously provided by Dr. Fumiko Sekiguchi (Kindai University) and maintained in Dulbecco’s Modified Eagle’s medium (DMEM; Nacalai Tesque Inc.) including 5.5 mM glucose and 1.1 mM sodium pyruvate, supplemented with 5% fetal bovine serum (FBS; Thermo Fisher Scientific Inc.) and Antibiotic-Antimycotic Mixed Solution (100 units/mL penicillin, 100 μg/mL streptomycin; Nacalai Tesque). ND7/23 cells were cultured in DMEM containing 5 or 60 mM glucose and 0.1, 1, or 10 mM sodium pyruvate supplemented with 1% FBS for 2 days on a 96-well clear plate (Thermo Fisher Scientific Inc.). To induce differentiation, ND7/23 cells were exposed to 5 μM Y27632 (FUJIFILM Wako Pure Chemical Corp., Osaka, Japan) and 1% nonessential amino acids (FUJIFILM Wako Pure Chemical Corp.). In an intervention study, ND7/23 cells were differentiated under normal conditions and then exposed to 60 mM glucose.

### 4.6. Neurite Outgrowth

After differentiation, the cells were fixed with 4% paraformaldehyde for 10 min at room temperature. After washing with PBS, the cells were immersed in blocking buffer. The cells were then reacted overnight at 4 °C with mouse anti-βIII tubulin monoclonal antibody (1:1000, Sigma–Aldrich Co. LCC, St. Louis, MO, USA) diluted in blocking buffer. After washing with PBS, the cells were incubated for 1 h at 37 °C with horseradish peroxidase conjugated-anti-mouse IgG antibody (1:200; Thermo Fisher Scientific Inc., Waltham, MA, USA) diluted in PBS. The signals were visualized using 0.01% diaminobenzidine tetrahydrochloride (DAB, Wako Co., Tokyo, Japan) and 0.01% hydrogen peroxide in PBS, and the cells were mounted with a mounting medium (SouthernBiotech, Birmingham, AL, USA). Images were captured using a microscope (BZ-X800, Keyence, Osaka, Japan). Neurite lengths were measured using WinROOF2015 (Mitani Corporation, Tokyo, Japan).

### 4.7. Measurement of ROS Production and Mitochondrial Membrane Potential 

Following the culture of ND7/23 cells, ROS production and mitochondrial membrane potential were assessed using the ROS Assay Kit—Highly Sensitive DCFH-DA—(Dojindo, Kumamoto, Japan) and the JC-10 Mitochondrial Membrane Potential Assay Kit—Microplate (Abcam, Cambridge, UK), respectively, according to the manufacturers’ instructions. The images were observed using a microscope (BZ-800) and signal intensity were quantified using ImageJ software, version 1.54f.

### 4.8. Statistical Analysis 

Data from animal studies and cultured cells are presented as the mean ± standard error of the mean (SEM) or standard deviation (SD). All statistical analyses were conducted using Easy R (EZR) [44]. Statistical analysis was performed using the student’s *t* test, and one-way analysis of variance (ANOVA), followed by post hoc comparisons with the Tukey HSD test, or *Kruskal-Willis* test, followed by post hoc comparisons with the Steel-Dwass test. A *p*-value < 0.05 between groups was considered statistically significant.

## Data Availability

All data presented in this paper are available in the manuscript or from the corresponding author (H. Y.).
